# Pedigree Investigation of Polish Sport Horses in Show Jumping: Insights for Global Breeding

**DOI:** 10.3390/ani16081152

**Published:** 2026-04-10

**Authors:** Tomasz Próchniak

**Affiliations:** Institute of Biological Basis of Animal Production, University of Life Sciences in Lublin, Akademicka 13, 20-950 Lublin, Poland; tomasz.prochniak@up.edu.pl; Tel.: +48-814456757

**Keywords:** Polish sport horse, pedigree analysis, inbreeding, genetic diversity, sport horse breeding

## Abstract

The aim of this study was to characterise the pedigree and genetic structure of Polish Sport Horses competing in Grand Prix show jumping events and to assess the implications for international sport horse breeding. Pedigrees of 513 horses were analysed, encompassing a total of 18,836 individuals over a maximum of 16 generations. The completeness and depth of the pedigrees allowed for a reliable estimation of inbreeding coefficients and genetic diversity. The mean inbreeding coefficient was low (0.645%), yet 82% of the horses exhibited some degree of inbreeding. The greatest loss of genetic variability was observed in non-founder generations, most likely due to the intensive use of a limited number of high-value stallions with domestic mares—a bottleneck effect. The most significant founders contributing to the population were the Thoroughbred stallions Ladykiller and Rantzau, as well as the Anglo-Arab stallion Ramzes, highlighting the international influence on the contemporary population. These findings emphasise the need for systematic monitoring of genetic diversity and the strategic use of pedigree data to minimise inbreeding and preserve the genetic potential of Polish Sport Horses for international breeding programmes.

## Simple Summary

Sport horses used in show jumping are bred to achieve high performance, but intensive selection and the repeated use of a limited number of stallions may reduce genetic diversity. This study analysed the pedigrees of horses competing in top-level national show jumping competitions in Poland to better understand their genetic background. We examined over 500 horses and their ancestors across many generations to evaluate relatedness and the level of inbreeding. The results showed that most horses were related to each other to some extent, although the overall level of inbreeding remained relatively low. A large proportion of the genetic contribution came from a small number of well-known stallions, which reflects common breeding practices in sport horse populations. These findings indicate that while current breeding strategies successfully produce competitive horses, they may also lead to a gradual loss of genetic diversity over time. Maintaining genetic variation is important for the long-term health, performance, and sustainability of horse populations. Therefore, monitoring pedigrees and using a wider range of breeding animals may help reduce the risk of excessive relatedness in future generations.

## 1. Introduction

The Polish Sport Horse (SP) is the most popular Polish sport horse breed, widely used both domestically and internationally. Although the breed has its own studbook and breeding programme, it comprises a population of animals with diverse genotypes and phenotypes [1], often resulting from crosses between Polish mares and recognised stallions of Belgian, Dutch, German, and French breeds. The breed’s studbook was established in 1977 and currently contains the largest active registered population within the Polish Horse Breeders Association. As of 2024, the active population comprised 1716 mares, 207 stallions, and 1956 foals, that underwent breeding inspections in that year [2]. On the international stage, horses registered in the SP studbook, similar to those in other Polish half-bred studbooks affiliated with the World Breeding Federation for Sport Horses, are designated with the PZHK abbreviation.

As with most sport horse breeding associations, the primary objective is the improvement of sport performance traits. Horses are bred to compete internationally across various equestrian disciplines. It should be noted that, in modern horse breeding, breed affiliation is typically determined by entry into a specific studbook rather than the possession of a particular set of inherited genes. The importation of genetic progress through the exchange of recognised stallions across breeds is common among breeding associations focused on producing riding horses. Consequently, sport horses constitute a distinct population, where individuals, although belonging to different breeds and studbooks, are interconnected through kinship. Furthermore, the performance value of these horses is evaluated under comparable environmental conditions, in training centres, young horse championships, and competitive events. Analysis of the breed composition of horses competing in show jumping indicates a declining representation of native breeds, whose selection for sport performance is less intensive [3,4].

Although the literature on sources of variation in selected traits and the assessment of performance and breeding value in show jumping horses is extensive [5,6,7,8], continuous updating of this knowledge is required. A valuable complement to such studies is the analysis of the dynamically changing genetic structure within the show jumping horse population and the assessment of the genetic consequences of breeding practices. Intensive selection aimed at improving riding traits usually leads to a reduction in genetic diversity, associated with a gradual increase in inbreeding. Numerous reports exist concerning inbreeding levels in various horse populations, including both sport and conservation herds [9,10,11,12]. Monitoring inbreeding is crucial to maintain genetic variability at acceptable levels [13,14,15], prevent inbreeding depression, and enable consistent breeding progress.

Regarding competition participation, show jumping is the most popular equestrian discipline in Poland and worldwide. Horses of various breeds, with seemingly diverse pedigrees, compete in this discipline. In Poland, the most challenging domestic competitions are the Final Grand Prix rounds [4]. Technical requirements for these courses are defined by the Polish Equestrian Federation, and the limited variation in course design renders the results from these events suitable for population studies. Jump heights are consistently 140–145 cm, depending on the prize pool for the competition. These events likely attract the best national horses, whose next level of competition is at international events. Moreover, competition results serve as indicators of performance value and may form the basis for inclusion in the studbook. Analysis of the genetic structure of Polish Sport Horses competing in Grand Prix competitions is therefore justified, given their prospective active use in breeding.

Previous pedigree studies of horses have not comprehensively covered the population of Polish Sport Horses and have often been limited to general inbreeding indicators. There is also a lack of detailed genetic analyses of horses actively competing in top-level events, such as Grand Prix. With the growing importance of international breeding, it is essential to understand which genetic lines dominate sport horse populations used both domestically and abroad, and what the implications are for genetic diversity. In light of the ongoing globalization of horse breeding and the gradual loss of genetic variability, conducting a study on the pedigrees and genetic structure of the largest breed group of Polish Sport Horses competing in Grand Prix events is fully justified.

The aim of this study was to characterise the pedigree and genetic structure of Polish Sport Horses used in show jumping and to assess the implications for international sport horse breeding.

## 2. Materials and Methods

Pedigree data for 513 Polish Sport Horses (283 males and 230 females) competing in Grand Prix Final competitions between 2020 and 2025 were analysed. These were all the horses participating in these competitions for which complete pedigree information was obtained for at least five generations. The pedigrees encompassed 18,836 individuals, reaching a maximum of 16 generations, with an average depth of seven generations. The studied horses were born between 1991 and 2018 and trained in equestrian centres across Poland. Polish Sport Horses represented approximately 17% of all competitors, forming the largest breed group in the analysed events.

Pedigree completeness was assessed using the discrete generation equivalent (g_e_) index [16] and the pedigree completeness index (C_p_) for five generations, as proposed by Cassell et al. [17]. The g_e_ index took into account the effective number of generations that contributed to the actual genetic diversity in the studied population, considering the relationships among ancestors, whereas C_p_ indicated, as a percentage, how many ancestors were known within a given number of generations. Using both indices allowed for a more comprehensive assessment of pedigree data quality and their suitability for inbreeding and genetic diversity analyses.

The contribution of individuals from different breed-origin groups in the three generations of ancestors was also determined. Five ancestral breed-origin groups were distinguished: Polish breeds, German breeds, Belgian and Dutch breeds, French breeds, and Thoroughbred horses. When defining the breed-origin groups, genetic similarity between breeds and their country of origin was taken into account. Due to smaller numbers, Belgian and Dutch horses were combined into a single group.

Individual inbreeding coefficients (F_i_) were calculated using the algorithm of Colleau [18], presented in an additive relationship matrix to assess homozygosity levels. Individual inbreeding increments (ΔF_i_) were calculated following Gutiérrez et al. [19]. Effective population size N_e_ was estimated based on the mean inbreeding increment (ΔF) according to Cervantes et al. [20], representing the size of a theoretical ideal population losing heterozygosity at the same rate as the observed population. Effective population size depended on reproductive structure, offspring number, fluctuations in cohort size, and overlapping generations.

Founder equivalents (f_e_) were calculated according to Lacy [21], representing the number of founders with equal genetic contribution that explain the observed genetic diversity. Founder genome equivalents (f_ge_), reflecting the minimal number of founders required to achieve the observed genetic diversity while accounting for genetic drift and bottlenecks, were additionally calculated [21]. The effective number of non-founders (N_enf_) was determined following Caballero and Toro [22], describing the genetic diversity loss due to drift in non-founder generations.

Genetic diversity (GD) was estimated using Lacy’s method [21,23]. The index 1-GD reflects the loss of genetic diversity relative to the founder generation due to drift and bottleneck effects, while 1-GD* quantifies diversity loss from unequal founder contributions [22]. The difference between GD* and GD indicates diversity loss due to drift in non-founder generations.

Founder allele contributions to mean inbreeding and coancestry were estimated using Sargolzaei and Colleau’s [24] method. Three ancestor contribution vectors were determined: m (Mendelian sampling variance), u (common ancestor contribution, identifying individuals contributing to inbreeding), and v (founder gene contribution).

Pedigree analyses were performed using the CFC 1.0 software [25]. Functions were applied to calculate individual inbreeding coefficients, coancestry, probabilities of gene origin, and to analyze the pedigree structure. Two grouping approaches were considered in the analysis: the first referring to the reference population, and the second to ancestral-breed groups. Default program settings were used, and individuals of unknown origin were treated as founders (generation 0). The applied mathematical formulas are presented in Table 1.

As the data were collected exclusively during official sport competitions and no procedures affecting animal welfare were conducted, the study complied with relevant legislation (Directive 2010/63/EU of the European Parliament and of the Council of 22 September 2010 on the protection of animals used for scientific purposes) [26].

## 3. Results

All horses in the reference population had both parents known, and their pedigrees encompassed a total of 18,836 individuals, including 640 founders with unknown parentage. The longest ancestral path (LAP) reached 16 generations (Table 2). The mean discrete generation equivalents in the reference population were 5.75 (range 2.71–7.53). The mean pedigree completeness for five generations of ancestors was very high at 98.45%.

Analysis of breed contributions in three generations of ancestors (Table 3) revealed the greatest representation of German breeds (42%), followed by Polish breeds (25%) and Belgian and Dutch breeds (20%). Furthermore, nearly 58% of the horses were sired by Belgian and Dutch stallions mated predominantly with Polish mares (SP, Małopolska, and Wielkopolska breeds). In the second and third ancestor generations, animals of German breeds, primarily Holstein, Hanoverian, and Westphalian, predominated on both paternal and maternal sides. Pedigrees also revealed a notable contribution of Thoroughbreds, accounting for approximately 12% in the third generation.

It was observed that 420 individuals (82%) exhibited non-zero inbreeding coefficients (Table 2). The mean inbreeding coefficient was 0.645%, with a maximum of 5.259%. The mean coancestry coefficient was just under 2%. The mean individual inbreeding increment (ΔF_i_) was 0.1%. Based on ΔF_i_, the realised effective population size (N_e_) was estimated at 395.

Table 3 also presents genetic diversity parameters calculated from the probability of allele origin in the reference population. The effective number of founders (f_e_) was 131, whereas the founder genome equivalent (f_ge_) was 26. The effective number of non-founders (N_enf_) was 32, markedly lower than f_e_.

The loss of genetic variability resulting solely from unequal founder contributions was almost five times lower than the total loss of genetic diversity, which also included the effects of genetic drift and population bottlenecks.

The genes of the 15 founders listed in Table 4 accounted for 25% of the population, explaining approximately 40% of the observed inbreeding. The top three stallions—Ladykiller, Rantzau, and Ramzes—contributed 7%, 8%, and 5%, respectively, to the actual inbreeding levels in the reference population.

## 4. Discussion

According to Cassell et al. [17], accurate and complete pedigrees provide reliable estimates of genetic variability within a population. The mean number of discrete generation equivalents (g_e_ = 5.75; Table 2) indicates satisfactory pedigree completeness and allows credible assessment of homozygosity. This value is comparable to results obtained by Cervantes et al. (2008) for Spanish Arabian horse pedigrees [20]. Similarly, the pedigree completeness index for five generations (C_p_ = 98.45%) [17] aligns with previous studies [27].

Coancestry and inbreeding coefficients were similar to those reported by Borowska and Szwaczkowski [9] for Polish horses assessed during performance tests in training centres. Although inbreeding occurs across various animal populations, the mean level in Polish Sport Horses was only 0.645%, not exceeding 5.259%. In comparison, higher mean inbreeding levels have been reported in native Italian horses (2.9%) [28], Brazilian sport horses (3.3%) [29], and Lusitano horses, where the mean inbreeding reached 11% [30]. In general, inbreeding levels in horse populations are considered less problematic than in other livestock species, such as dairy cattle. Nevertheless, 82% of the SPs showed some degree of inbreeding. With the growth of equestrian sport, stricter selection criteria may lead to partial loss of genetic diversity in the SP population, similar to that observed in other horse populations and breeds.

The loss of genetic diversity due solely to unequal founder contributions (1-GD*) was considerably lower than the total loss, which also included genetic drift and bottleneck effects (1-GD). This likely reflects the limited number of stallions used in certain periods, whose numerous offspring were heavily utilised in sport horse breeding—a common phenomenon in sport horse populations. In recent decades, the importation of genetic progress has been facilitated by advances in reproductive technologies, resulting in frequent mating of domestic mares with recognised foreign stallions. In the studied SPs, there was a substantial contribution of ancestors from German, Belgian, Dutch, and French breeds (Table 3). Strategic breeding increases the pool of desirable alleles but carries a risk of inbreeding and bottleneck effects, potentially leading to significant loss of genetic variability. Bottleneck effects have also been observed in Hucul horses [31], which are not selected for sport performance and maintain a relatively large population in Poland and Slovakia.

The observed phenomena indicate the need for continuous monitoring of the pedigree structure and the rate of genetic variability loss in Polish Sport Horses. The results also emphasize the importance of population monitoring through regular assessment of inbreeding coefficients, planned matings, and maintaining an adequate number of breeding animals from diverse genetic lines. These data should be made available to breeders already at the stage of performance evaluation of young horses. Such measures can help mitigate the effects of genetic drift and population bottlenecks, supporting the long-term maintenance of genetic diversity within the population.

Genetic diversity parameters (Table 2), based on allele origin probabilities, describe the expected heterozygosity and are widely used even with limited population sizes and few known generations [32]. Based on f_e_ and f_ge_, SP breeding preferentially uses a narrow group of stallions. The effective number of non-founders (N_enf_) being higher than f_ge_ indicates accumulation of genetic drift in non-founder generations. The number of founders in the pedigrees exceeding the effective number of founders and founder genomes is likely due to the fact that Polish Sport Horses often have native breed ancestors, such as Małopolska and Wielkopolska horses, in their pedigrees. Although the number of these ancestors was considerable, their genetic contribution to the current population is relatively small compared to the contribution of internationally recognized elite sires, who, in turn, descend from a limited number of founders. This pedigree structure indicates an unequal genetic contribution of individual founders, which is important for maintaining the genetic diversity of the population.

The highest contributions to the studied population (Table 4) came from the Thoroughbred stallions Ladykiller and Rantzau, as well as the Polish-bred Anglo-Arab stallion Ramzes. High contributions of these stallions in contemporary sport horse populations have been confirmed in Poland [9], Brazil [29] and Germany [33]. Ladykiller contributed over 4% to the population, and his offspring, including Landgraf I, were widely used internationally. Ramzes is a founder of the German sport horse line, giving rise to horses such as Ramiro Z and Ratina Z, also used in Belgian Zangersheide breeding—one of the most prominent show jumping breeds.

## 5. Conclusions

Pedigree completeness and depth were sufficient to reliably assess genetic diversity in Polish Sport Horses. Most genetic variability loss occurred in non-founder generations, likely due to mating domestic mares with a limited number of high-value foreign stallions, whose numerous offspring were intensively used—a typical bottleneck effect.

The greatest genetic contributions to the studied population were from the Thoroughbred stallions Ladykiller and Rantzau, and the Anglo-Arab stallion Ramzes, confirming previous observations that Polish Sport Horses descend from a limited number of recognised stallions, whose progeny have been widely utilised in breeding.

Although the mean inbreeding coefficient was at an acceptable level, it is concerning that 82% of the studied horses exhibited some inbreeding. Combined with the limited number of stallions used, this may lead to a rapid reduction in genetic variance in the future.

Consequently, the genetic diversity of Polish Sport Horses should be systematically monitored, and pedigree information should inform breeding decisions to minimise the risk of diversity loss and bottleneck effects.

## Figures and Tables

**Table 1:** Mathematical formulas of the estimated parameters.

Parameter	Formula	Formula Description
Discrete generation equivalents (g_e_)	$g_{e}=\frac{1}{N}\sum_{j=1}^{N}\sum_{i=1}^{n}\frac{1}{2^{g_{ij}}}$	n_j_—number of known ancestors of the j-th individual g_ij_ is the number of generations between the i-th ancestor and the j-th animal N—number of animals in the reference population
Pedigree completeness Cassel method (C_p_)	$C_{p}=\frac{a_{k}}{\sum_{i=1}^{g}2^{i}}$	a_k_—number of known ancestors in the k-th generation, g—number of generations, 2^i^—maximum number of ancestors in the i-th generation
Individual increase in inbreeding (ΔF_i_)	$∆F_{i}=1-\sqrt[{g_{e}-1}]{1-F_{i}}$	F_i_—individual inbreeding coefficient g_e_—discrete generation equivalent.
Effective population size (N_e_)	$N_{e}=\frac{1}{2∆F}$	ΔF—individual inbreeding gain
Founder equivalent (f_e_)	$f_{e}=\frac{1}{\sum \left(p_{i}^{2}\right)}$	p_i_—proportion of genes of the i-th founder in the reference population.
Founder genome equivalent (f_ge_)	$f_{ge}=\frac{1}{\sum \left(\frac{p_{i}^{2}}{r_{i}}\right)}$	p_i_—proportion of genes contributed by the i-th founder to the offspring population, r_i_—proportion of genes of the i-th founder that are preserved in the reference population.
Non-founder equivalent (N_enf_)	$N_{enf}={\left(\frac{1}{f_{ge}}-\frac{1}{f_{e}}\right)}^{-1}$	f_ge_—founder genome equivalent f_e_—founder equivalent
Genetic diversity (GD) unequal founder contribution, bottlenecks, and genetic drift	$GD=1-\frac{1}{{2f}_{ge}}$	f_ge_—founder genome equivalent
Genetic diversity (GD*) only unequal founder contribution	${GD}^{*}=1-\frac{1}{{2f}_{e}}$	f_e_—founder equivalent
Contribution of founders’ genes (c_y_)	$c_{y}=\sum_{j}p_{yj}\times u_{j}$	p_yj_—proportion of genes of founder (y) transmitted through Nodal Common Ancestor (j) u_j_—contribution of genes of Nodal Common Ancestor (j)
Contribution of founders’ genes to average inbreeding (v1_y_)	${v1}_{y}=\frac{1}{N}\sum_{i=1}^{N}c_{y,i}$	c_y,i_—contribution of founder (y) to the inbreeding of individual (i) N—number of individuals in the population
Contribution of founders’ genes to average coancestry (v2_y_)	${v2}_{y}=\frac{1}{N^{2}}\sum_{i=1}^{N}\sum_{j=1}^{N}c_{y,i}\times c_{y,j}$	c_y,i_,c_y,j_—contributions of founder (y) to individuals (i) and (j), respectively
Contributions of Mendelian sampling variance of ancestors (m)	$F_{i}=\sum_{k}m_{ik}$	F_i_—inbreeding coefficient of individual (i) m_ik_—contribution of the Mendelian sampling variance of ancestor (k) to F_i_
Contributions of genes of nodal common ancestors (u)	$F_{i}=\sum_{j}u_{ij}$	u_ij_—contribution of genes of Nodal Common Ancestor (j) to the inbreeding of individual (i)

**Table 2:** Pedigree structure, homozygosity characteristics, and loss of genetic diversity in the population of Polish Sport Horses.

Parameter	Symbol	Value
Pedigree structure		
Size of the reference population		513
Number of individuals in the pedigree		18,836
Number of founders in the population		640
Longest ancestral path	LAP	16
Pedigree completeness (%)	C_p_	98.45
Average number of discrete generation equivalents	g_e_	5.753
Minimum number of discrete generation equivalents	g_e_ min	2.711
Maximum number of discrete generation equivalents	g_e_ max	7.525
Homozygosity in the population		
Number of inbreds		420
Average inbreeding coefficients (%)	$\bar{F}$	0.645
Average inbreeding coefficients in the inbreds (%)	$\bar{F_{i}}$	0.768
Minimum of inbreeding coefficients (%)	F_min_	0.003
Maximum of inbreeding coefficients (%)	F_max_	5.259
Average individual increase in inbreeding (%)	ΔF_i_	0.100
Effective population size	N_e_	395.144
Average coancestry (%)	$\bar{f}$	1.935
Genetic diversity in the population		
Effective number of founders	f_e_	131.083
Effective number of founder genomes	f_ge_	25.841
Effective number of non-founders	N_enf_	32.186
Loss of genetic diversity (unequal founder contribution, bottlenecks, and genetic drift)	1-GD	0.019
Loss of genetic diversity (only unequal founder contribution)	1-GD*	0.004

**Table 3:** Origin of 513 Polish Sport Horses, including three generations of ancestors.

Breeding Group	1st Generation		2nd Generation		3rd Generation		Average
	♂♂	♀♀	♂♂	♀♀	♂♂	♀♀	
Polish breeds	0.36	0.58	0.05	0.31	0.07	0.13	0.25
German breeds	-	0.23	0.64	0.41	0.63	0.61	0.42
Belgian and Dutch breeds	0.58	0.10	0.17	0.13	0.06	0.15	0.20
French breeds	0.06	0.04	0.11	0.03	0.13	0.06	0.07
Thoroughbred	-	0.05	0.03	0.12	0.11	0.05	0.06

**Table 4:** Most important founders in the tested population.

Founder Breed Number	Sex ^1^	Breed ^2^	Birth Year	c_y_ [%]	v1_y_ [%]	m1_y_ [%]	u1_y_ [%]	v2_y_ [%]	m2_y_ [%]	u2_y_ [%]
Ladykiller DE306064000861	S	xx	1961	4.031	0.068	0.044	0.026	0.142	0.081	0.062
Rantzau DE306064779046	S	xx	1946	3.172	0.080	0.045	0.019	0.132	0.050	0.017
Ramzes DE321210365437	S	xo	1937	3.075	0.054	0.039	0.032	0.106	0.047	0.037
Cottage Son DE321210375544	S	xx	1944	2.934	0.055	0.041	0.035	0.097	0.043	0.037
Loretto DE321210289432	S	hol	1932	2.171	0.033	0.018	0.016	0.063	0.024	0.021
Furioso DE306064703339	S	xx	1939	2.074	0.033	0.014	0.010	0.068	0.022	0.015
Fougere DE304048545371	M	sf	1971	1.375	0.000	0.000	0.000	0.033	0.009	0.000
Vestale du Bois 25000149001674K	M	sf	1942	1.242	0.030	0.006	0.000	0.049	0.008	0.000
Anblick DE321210369138	S	xx	1938	1.061	0.017	0.006	0.003	0.027	0.006	0.003
Dame de Ranville DE304046020147	M	sf	1947	1.016	0.000	0.000	0.000	0.020	0.005	0.001
Makler I DE321210284029	S	hol	1929	0.841	0.011	0.003	0.002	0.020	0.004	0.003
Manometer DE321210379553	S	xx	1953	0.788	0.005	0.001	0.000	0.018	0.003	0.002
Fanatiker DE321210321940	S	hol	1940	0.778	0.007	0.002	0.002	0.017	0.003	0.002
Duellant DE331310358643	S	han	1943	0.714	0.003	0.003	0.003	0.012	0.003	0.002
Ferdinand DE331310340641	S	han	1941	0.688	0.000	0.000	0.000	0.012	0.002	0.002

^1^ S—sire, M—mare; ^2^ xx—thoroughbred, sf—selle francais, xo—halfbred Anglo-Arabian horse, hol—Holstein horse, han—Hanoverian horse. c_y_—contribution of founders’ genes; v1_y_—contribution of founders’ genes to the average inbreeding coefficient; v2_y_—contribution of founders’ genes to the average coancestry; m—contributions of Mendelian sampling variance of ancestors; u—contributions of genes of nodal common ancestors.

## Data Availability

The data used in this study are the property of the author and can be made available upon reasonable request by contacting the corresponding author.

## Abbreviations

The following abbreviations are used in this manuscript:

C_p_	Pedigree completeness index
$\bar{F}$	Mean inbreeding coefficient
F_i_	Individual inbreeding coefficient
GD	Genetic diversity
GD*	Genetic diversity accounting for unequal founder contribution
g_e_	Discrete generation equivalents
LAP	Longest ancestral path
N_e_	Effective population size
N_enf_	Effective number of non-founders
f_e_	Founder equivalents
f_ge_	Founder genome equivalents
SP	Polish Sport Horse
ΔF_i_	Individual increase in inbreeding
